# Genome Sequence of Bacillus halotolerans Strain MS50-18A with Antifungal Activity against Phytopathogens, Isolated from Saline Soil in San Luís Potosí, Mexico

**DOI:** 10.1128/genomeA.00135-18

**Published:** 2018-03-08

**Authors:** Jaime Sagredo-Beltrán, Yumiko De La Cruz-Rodríguez, Miguel Alvarado-Rodríguez, Julio Vega-Arreguín, Raúl Rodríguez-Guerra, Alejandro Alvarado-Gutiérrez, Saúl Fraire-Velázquez

**Affiliations:** a Biología Integrativa de Plantas y Microorganismos, Unidad Académica de Ciencias Biológicas, Universidad Autónoma de Zacatecas, Zacatecas, Mexico; b Unidad Académica de Agronomía, Universidad Autónoma de Zacatecas, Zacatecas, Mexico; c ENES-León, Universidad Nacional Autónoma de México, León, Guanajuato, Mexico; d Campo Experimental General Terán, INIFAP, General Terán, Nuevo León, Mexico

## Abstract

Bacillus halotolerans strain MS50-18A, isolated from saline soil, possesses antifungal activity toward root rot causal phytopathogens and has friendly interactions with the chili pepper plant. The draft genome sequence is 4.06 Mb in length and contains 4,215 genes. Genes related to glycine/betaine uptake and bacilysin biosynthesis are present, supporting its saline stress tolerance and antifungal activity.

## GENOME ANNOUNCEMENT

Bacterial isolates with inhibitory activity against pathogens of horticultural crops that are able to establish friendly interactions with the host plants of the same crops could offer more possibilities to cope with phytopathogens. A nonpathogenic interaction occurring between a bacterial isolate and a host plant can trigger the induced systemic resistance (ISR) in the plant (1), improving the biocontrol capacity of the bacterium against intruder undesirable microorganisms (2). Brevibacterium halotolerans (3) is a species name still in debate in relation to heterotypic synonyms (4–6), with a recently suggested reclassification of Bacillus halotolerans (6). From a study of soil samples from agricultural and forest areas in Zacatecas and San Luis Potosí, Mexico, in which we aimed to isolate bacterial strains with the capability to inhibit root rot causal phytopathogens, namely Phytophthora capsici, Fusarium solani, Rhizoctonia solani, and Fusarium oxysporum, we isolated Bacillus halotolerans strain MS50-18A. In dual confrontation using tryptic soy agar (TSA), King’s B medium (KB), or peptone-dextrose agar (PDA) solid medium, the MS50-18A strain prevents the growth of these four phytopathogens at least to 60% inhibition; furthermore, it exhibits a friendly interaction with pepper plantlets when inoculated in the root and evaluated for 2 weeks in pots or under *in vitro* conditions. Moreover, this Bacillus halotolerans strain is a producer of the auxin-related phytohormone indoleacetic acid. To better characterize this bacterium, we sequenced its genome using a MiSeq sequencer (Illumina) in a 2 × 75 paired-end run. To assemble the genome, the SPAdes genome assembler (7) was used, and the quality of the assembly was analyzed using QUAST (8). A total length of 4.069 Mb of draft genome sequence was obtained in 44 contigs and with a G+C content of 43.76%. The genome annotation was achieved using the NCBI Prokaryotic Genome Annotation Pipeline (9). In the sequenced genome, a total of 4,215 genes, 3,998 coding genes, 87 RNA genes, 7 5S, 2 16S, and 1 23S rRNAs, and 130 pseudogenes were revealed. In relation to bacterial response to osmotic stress, it is well known that the uptake of glycine/betaine from the extracellular space through the high-affinity uptake system is a mechanism by which bacterial cells overcome the saline stress, with glycine/betaine acting as osmoprotectants (10, 11); in the genome of this B. halotolerans strain, two glycine/betaine ABC transporters, two glycine/betaine ABC transporter ATP-binding proteins, and a glycine/betaine ABC transporter permease were found, suggesting that this system probably functions in this bacterium to accumulate solutes for osmoregulation. This MS50-18A strain contains also a *bacA* gene that codes for an enzyme involved in the biosynthesis of bacilysin (12), a non-ribosomally synthesized dipeptide that is processed by a peptidase, resulting in l-anticapsin, which inhibits the glucosamine synthase that causes lysis of fungal cells (13); this could explain the ability of this bacterium to inhibit fungal phytopathogens. Further microbiological confrontation studies and evaluations of plant protection in other plant pathosystems, in addition to salt stress assays, will assess the full biotechnological usefulness of this bacterium.

### Accession number(s).

This whole-genome shotgun project has been deposited at DDBJ/ENA/GenBank under the accession number MLCY00000000. The version described in this paper is version MLCY01000000.
